# Incidence and risk factors of colorectal delayed post‐polypectomy bleeding in patients taking antithrombotics

**DOI:** 10.1111/1751-2980.13034

**Published:** 2021-08-16

**Authors:** Zhen Yan, Feng Gao, Jiang Xie, Jie Zhang

**Affiliations:** ^1^ Department of Gastroenterology Beijing Anzhen Hospital, Capital Medical University Beijing China; ^2^ Department of Respiratory and Critical Medicine Beijing Anzhen Hospital, Capital Medical University Beijing China

**Keywords:** antithrombotics, delayed postoperative bleeding, incidence, polypectomy, risk factors

## Abstract

**Objective:**

In this study we aimed to investigate the incidence and risk factors for delayed post‐polypectomy bleeding (DPPB) in Chinese patients taking antithrombotics including antiplatelet agents and anticoagulants.

**Methods:**

A retrospective study was conducted in patients who underwent colorectal polypectomy from January 2017 to May 2020. Their demographic characteristics, features of the polyps including number, size, morphology, and location, and use of antiplatelet agents and anticoagulants were collected. The incidence and risk factors for DPPB were compared between the patients with and without antithrombotic use.

**Results:**

A total of 5152 polyps from 2267 patients were resected under endoscopy. Of these patients, 35 (1.54%) experienced DPPB. Compared with the control group who did not take antithrombotics (1.18%), the incidence of DPPB was significantly higher in patients treated with heparin bridge (HB) therapy (17.39%; *P <* 0.001) and clopidogrel (4.88%; *P* = 0.022), but did not differ in patients taking aspirin (1.28%), dual antiplatelet therapy (3.70%), warfarin alone (0%), or direct oral anticoagulants (3.85%). Using the multivariate analysis, HB therapy (odds ratio [OR] 16.735, 95% confidence interval [CI] 4.320‐64.834, *P* < 0.001), male sex (OR 3.825, 95% CI 1.298‐11.265, *P* = 0.015), polyps >1 cm (OR 4.584, 95% CI 1.782‐11.794, *P* = 0.002) and rectal polyps (OR 8.820, 95% CI 3.968‐19.602, *P* < 0.001) were independently associated with a high risk of DPPB.

**Conclusions:**

HB and clopidogrel therapies significantly increase the incidence of DPPB. HB therapy, male sex, polyp size and polyps located in the rectum are significant risk factors for DPPB.

## Graphical abstract

Patients on antithrombotics are at a higher risk of delayed post‐polypectomy bleeding (DPPB) compared with those who do not take antithrombotics. Patients who receive heparin bridge (HB) therapy or clopidogrel have a significantly higher incidence of DPPB than those who do not. Abbreviations: DAPT, dual antiplatelet therapy; DOAC, direct oral anticoagulants.

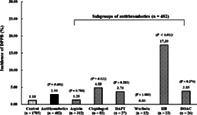

## 1 INTRODUCTION

Post‐polypectomy bleeding (PPB) is the main complication of endoscopic colorectal polypectomy. European, American and Asian guidelines have stated that polypectomy is a high‐risk endoscopic procedure due to the risk of postoperative bleeding.^1^, ^2^, ^3^ With the growing aging population, the incidence rates of cardiovascular and cerebrovascular diseases have been increasing, resulted in an increased use of antithrombotics (including antiplatelet agents and anticoagulants).^4^ Moreover, patients with ischemic heart disease have a 1.9‐fold higher risk of developing colonic polyps and colon cancer than the general population.^5^ Therefore, patients on antithrombotics have a greater need for endoscopic polypectomy than those who are not on antithrombotics. How to avoid the occurrence of PPB and to reduce the risk of thromboembolism caused by the discontinuation of antithrombotics is a dilemma faced by both gastroenterologists and cardiologists. So far there has been no standard periprocedural antithrombotic strategy.

The incidence and risk factors of delayed post‐polypectomy bleeding (DPPB) in patients treated with antithrombotics in China are under investigation; few studies on DPPB in Chinese patients have been published. The use of direct oral anticoagulants (DOAC) is increasing gradually; however, the relationship between DOAC and DPPB remains unclear. In this study, we aimed to analyze the rate of DPPB related to the administration of not only traditional antithrombotics but also new oral anticoagulants, and to report for the first time the correlation between antithrombotic therapy and DPPB in a group of Chinese patients on antithrombotics, and to evaluate its incidence and risk factors.

## 2 PATIENTS AND METHODS

### 2.1 Patient selection

This retrospective study was performed at Beijing Anzhen Hospital, Capital Medical University, one of the largest medical centers for cardiovascular diseases in Beijing, China. From January 2017 to May 2020, a total of 2710 patients underwent endoscopic colorectal polypectomy at our hospital. Patients at the age of 18 years old or elder who underwent energized (hot) procedures were included. The exclusion criteria were: (a) patients aged under 18 years; (b) underwent endoscopic submucosal dissection or non‐energized (cold) procedure; (c) polyp size <5 mm; and (d) insufficient patient data. The medical records of the patients and the polyps were obtained from the electronic medical record system and endoscopic database of the hospital. The following data were extracted: patient's age, sex, comorbidities (hypertension, diabetes mellitus, coronary heart disease, chronic kidney disease, chronic pulmonary disease, cerebrovascular disease, cirrhosis, peripheral arterial disease, pulmonary embolism, deep vein thrombosis, pacemaker implantation and malignancy), and their discontinuation and resumption of antithrombotic medications. The number, size, morphology and location of the polyps were recorded by reviewing the colonoscopy reports. Patients gave their written informed consent to undergo the endoscopic procedure and to their potential inclusion in the retrospective analysis afterwards. This study was approved by the Institutional Review Board of Beijing Anzhen Hospital (no: 2020070X).

**FIGURE 1:**
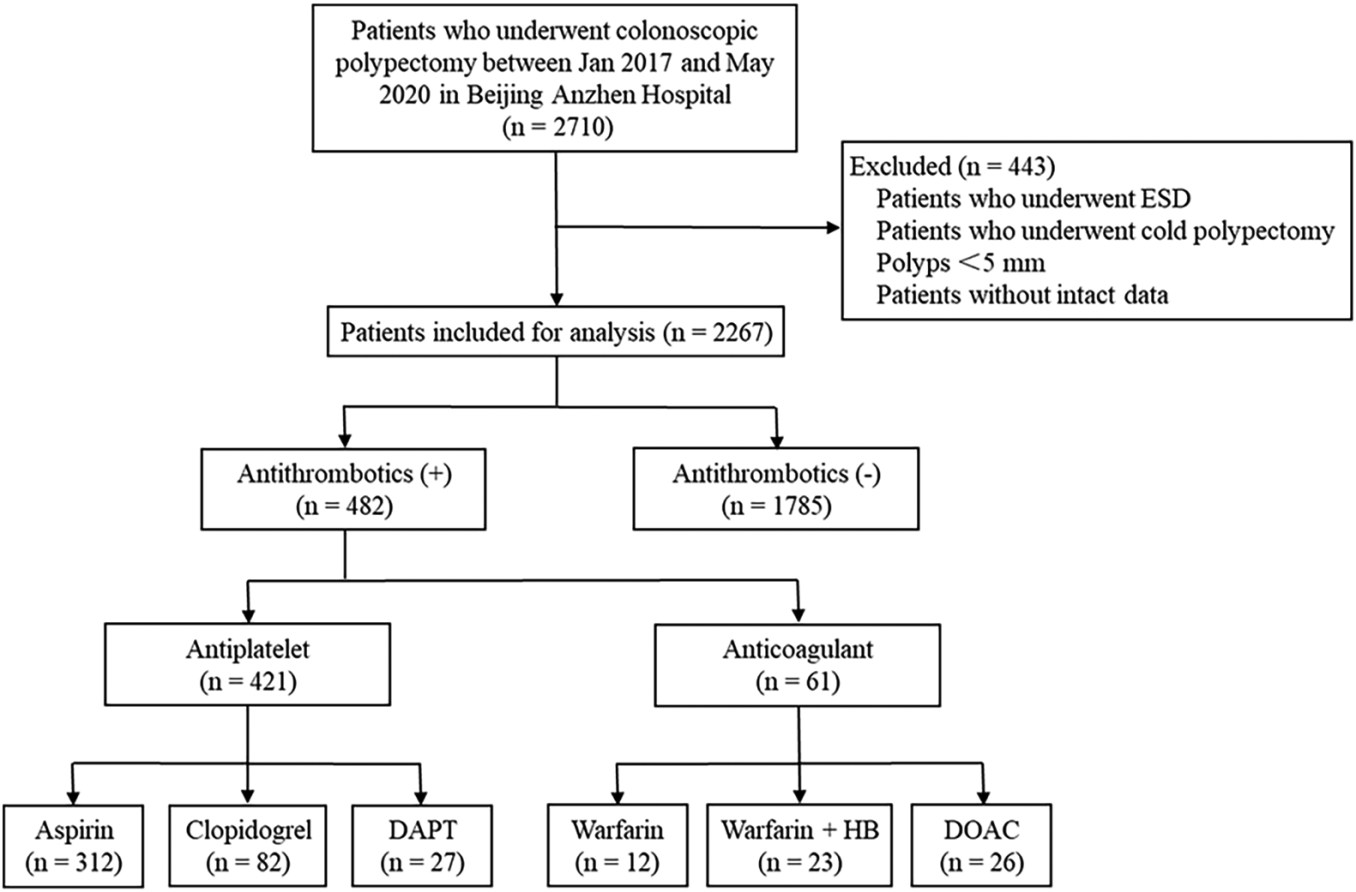
Flowchart of patient enrollment. Abbreviations: DAPT, dual antiplatelet therapy; DOAC, direct oral anticoagulants; ESD, endoscopic submucosal dissection; HB, heparin bridge

The patients were then divided into the non‐antithrombotic group and the antithrombotic group depending on their use of antithrombotic agents or not, and the former group served as the control group. Patients in the antithrombotic group were further divided into six subgroups according to the types of antithrombotic used: aspirin, clopidogrel, dual antiplatelet therapy (DAPT), warfarin, warfarin with heparin bridge (HB) therapy, and DOAC (including 16 rivaroxaban and 10 dabigatran agents). The flowchart of patient selection is shown in Figure 1.

### 2.2 Antithrombotics

Aspirin and clopidogrel (or ticagrelor) were suspended at 7 and 5 days, respectively, prior to the endoscopic procedure, until 48 hours after the procedure. Similarly, DOAC were discontinued at least 48 hours before endoscopy and resumed within 48 hours after the procedure. Warfarin was stopped at 5 days before endoscopy. Patients on warfarin were divided into a warfarin alone and a warfarin with HB therapy group according to their thromboembolic risk, as defined based on the European guidelines.^1^ In those at a low risk of thromboembolism, a polypectomy was performed when their international normalized ratio (INR) decreased to <1.5, and warfarin was resumed at the night of endoscopy. While in patients at a high risk of thromboembolism, low‐molecular‐weight heparin (LMWH) was started 2 days after warfarin was discontinued and stopped at least 24 hours before the endoscopic procedure. Warfarin was resumed at the night of endoscopic procedure and LMWH was continued until the INR returned to the therapeutic range (1.8‐2.5).

### 2.3 Endoscopic resection of the polyps

Colonoscopic polypectomy was performed using an electric endoscope (CF‐HQ290I or CF‐H260AI; Olympus, Beijing, China). Generally, polypectomy was performed by endoscopic mucosal resection or a hot snare polypectomy using an electrosurgical device (VI0200S; ERBE China, Shanghai, China). Prophylactic clipping was routinely performed after polyps larger than 5 mm were resected by using the hot polypectomy, and hemostatic clipping or thrombin spraying was considered when delayed bleeding occurred. All procedures were performed by five experienced endoscopists, each of whom had performed more than 10 000 colonoscopies.

### 2.4 End‐points

The primary end‐point of the study was DPPB, which was defined as overt melena or hematochezia occurring from 24 hours to 30 days after the polypectomy. Follow‐up visit was performed via telephone every other week after patient's discharge to ascertain whether melena or hematochezia occurred, which guaranteed their immediate referral to a gastrointestinal consultation. For those without DPPB, a postoperative evaluation was routinely arranged at 1 month after discharge. The secondary end‐points were interventional radiology or surgery, severe thromboembolic events, and death. Thromboembolic events were defined as stroke, transient ischemic attack, acute coronary syndrome, systemic embolism, pulmonary embolism or deep vein thrombosis.^6^

### 2.5 Statistical analysis

Statistical analyses were performed using the SPSS Statistic version 22.0 (IBM, Armonk, NY). Continuous variables were expressed as mean ± standard deviation and the differences between the two groups were analyzed by using an independent‐sample *t*‐test, whereas categorical variables were expressed as numbers and percentages and were compared by using the χ^2^ test or Fisher's exact test. A logistic regression analysis was performed to determine the risk factors associated with DPPB. Variables with a *P* value lower than 0.1 in the univariate analyses were included in the multivariate model. Odds ratio (OR) and 95% confidence interval (CI) were calculated to identify independent prognostic factors for DPPB. A two‐tailed *P* value of <0.05 was considered to indicate statistical significance.

## 3 RESULTS

### 3.1 Incidence of DPPB

**FIGURE 2:**
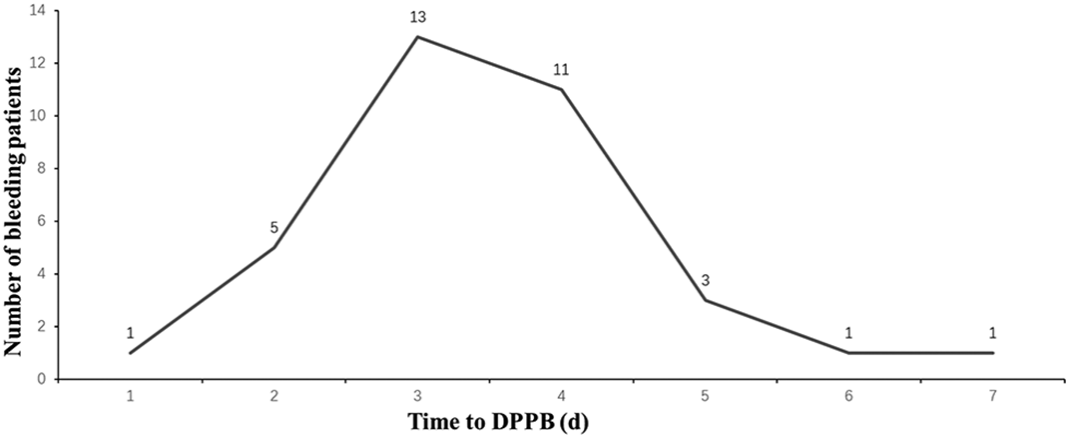
Time point of delayed post‐polypectomy bleeding (DPPB)

Among the 2710 patients who underwent endoscopic colorectal polypectomy from January 2017 to May 2020, 443 were excluded, and 2267 patients were finally enrolled in this study (Figure 1). Of them 1785 patients did not have antithrombotic administration and were included as the control group, whereas the other 482 patients were included as the antithrombtic group. A total of 5152 polyps were endoscopically resected. Altogether 35 patients experienced delayed bleeding after endoscopic resection and the incidence of DPPB was 1.54% (35/2267). The median time to postoperative occurrence of DPPB was 3 days (range 1‐7 d) and 30 (85.71%) patients experienced bleeding within 4 days (Figure 2).

The DPPB rate in the control group was 1.18% (21/1785). Taking antithrombotics was significantly associated with a higher postoperative bleeding rate (2.90% [14/482]) compared with the control group (*P* = 0.006). Compared with the control group, the rate of DPPB was comparable in the aspirin group (1.28%; *P* = 0.780) and the DAPT group (3.70%; *P* = 0.283), but was significantly higher in the clopidogrel group (4.88%; *P* = 0.022) and the warfarin with HB therapy group (17.39%; *P <* 0.001). No patients (0% [0/12]) in the warfarin alone group experienced DPPB. No patient received heparin among those taking DOAC and the DPPB rate of the DOAC group was comparable to that in the control group (3.85% vs 1.18%, *P* = 0.274) (Table 1).

**TABLE 1:** Incidence of delayed post‐polypectomy bleeding (DPPB)

	Bleeding (n)	No bleeding (n)	Incidence of DPPB (%)	*P* value^a^
Control (no antithrombotics)	21	1764	1.18	
Antithrombotics	14	468	2.90	0.006
Aspirin	4	308	1.28	0.780
Clopidogrel	4	78	4.88	0.022
DAPT	1	26	3.70	0.283
Warfarin	0	12	0	1.000
Warfarin + heparin bridge therapy	4	19	17.39	*<*0.001
DOAC	1	25	3.85	0.274

Abbreviations: DAPT, dual antiplatelet therapy; DOAC, direct oral anticoagulants.

^a^ χ^2^ test or Fisher's exact test compared with the control group.

### 3.2 Risk factors for DPPB

The characteristics of the patients who experienced DPPB and those who did not are summarized in Table 2. Compared with patients in the non‐bleeding group, those in the bleeding group there were significantly higher proportions of male subjects, those with polyps >1 cm and pedunculated and rectal polyps. There were no significant differences in age and comorbidities between the two groups (all *P* > 0.05).

**TABLE 2:** Characteristics of patients based on the occurrence of delayed post‐polypectomy bleeding

Variables	Bleeding (n = 35)	No bleeding (n = 2232)	*P* value^a^
Age, y (mean ± SD)	56.6 ± 13.3	59.8 ± 11.6	0.113
Male sex, n (%)	31 (88.6)	1404 (62.9)	0.002
Comorbidity, n (%)			
Hypertension	14 (40.0)	975 (43.7)	0.663
Diabetes mellitus	3 (8.6)	402 (18.0)	0.148
Coronary heart disease	7 (20.0)	290 (13.0)	0.210
PCI	4 (11.4)	124 (5.6)	0.132
CABG	1 (2.9)	22 (1.0)	0.302
Chronic kidney disease	0 (0)	27 (1.2)	1.000
Chronic pulmonary disease	1 (2.9)	102 (4.6)	1.000
Cerebrovascular disease	2 (5.7)	137 (6.1)	1.000
Cirrhosis	0 (0)	12 (0.5)	1.000
Peripheral arterial disease	1 (2.9)	13 (0.6)	0.196
Pulmonary embolism	1 (2.9)	9 (0.4)	0.144
Deep vein thrombosis	0 (0)	7 (0.3)	1.000
Pacemaker implantation	0 (0)	10 (0.4)	1.000
Malignancy, n (%)	1 (2.9)	135 (6.0)	0.720
Polyps, n (%)			
Number ≥3	12 (34.3)	741 (33.2)	0.892
Size >1 cm	27 (77.1)	710 (31.8)	<0.001
Pedunculated shape	21 (60.0)	456 (20.4)	<0.001
LST	2 (5.7)	158 (7.1)	1.000
Lesion in the right side colon	9 (25.7)	804 (36.0)	0.207
Lesion in the rectum	12 (34.3)	174 (7.8)	<0.001

Abbreviations: CABG, coronary artery bypass grafting; LST, laterally spreading tumor; PCI, percutaneous coronary intervention; SD, standard deviation.

^a^ Independent‐sample *t* test, χ^2^ test or Fisher's exact test, when appropriate.

In the univariate analysis (Table S1), male sex (OR 4.571, 95% CI 1.608‐12.994, *P* = 0.004), clopidogrel use (OR 4.308, 95% CI 1.444‐12.851, *P* = 0.009), and HB therapy (OR 17.684, 95% CI 5.539‐56.461, *P* < 0.001) were associated with a high risk of DPPB. Similarly, patients with polyps larger than 1 cm (OR 7.235, 95% CI 3.270‐16.005, *P* < 0.001), pedunculated polyps (OR 5.858, 95% CI 2.956‐11.610, *P* < 0.001) or polyps located in the rectum (OR 8.946, 95% CI 4.349‐18.402, *P* < 0.001) were related with a higher risk of DPPB.

The risk factors for DPPB identified in the multivariate analysis are summarized in Table 3. The prognostic values of the morphology of the polyps and clopidogrel treatment were not significant after adjusting for covariates, whereas HB therapy (OR 16.735, 95% CI 4.320‐64.834, *P <* 0.001), male sex (OR 3.825, 95% CI 1.298‐11.265, *P* = 0.015), polyp size of >1 cm (OR 4.584, 95% CI 1.782‐11.794, *P =* 0.002) and polyps located in the rectum (OR 8.820, 95% CI 3.968‐19.602, *P* < 0.001) were retained as independent risk factors for DPPB. In terms of antithrombotics, only the HB therapy group had a significantly higher risk of DPPB than the control group.

**TABLE 3:** Multivariate analysis of risk factors for delayed post‐polypectomy bleeding

Variables	OR (95% CI)	*P* value^a^
Sex		
Female (ref.)	1.000	
Male	3.825 (1.298‐11.265)	0.015
Polyp size		
≤1 cm (ref.)	1.000	
>1 cm	4.584 (1.782‐11.794)	0.002
Polyp morphology		
Non‐pedunculated (ref.)	1.000	
Pedunculated	2.220 (0.964‐5.111)	0.061
Polyp location		
Colon (ref.)	1.000	
Rectum	8.820 (3.968‐19.602)	<0.001
Antithrombotic used		
Control (ref.)	1.000	
Aspirin	1.112 (0.365‐3.388)	0.852
Clopidogrel	2.917 (0.860‐9.888)	0.086
DAPT	1.362 (0.140‐13.277)	0.790
Warfarin	NA	0.999
Warfarin + HB therapy	16.735 (4.320‐64.834)	<0.001
DOAC	2.110 (0.251‐17.728)	0.492

Abbreviations: CI, confidence interval; DAPT, dual antiplatelet therapy; DOAC, direct oral anticoagulants; HB, heparin bridge; NA, not applicable; OR, odds ratio; ref., reference.

^**a**^ Multivariate logistic regression analysis.

### 3.3 Emergency endoscopy for DPPB

All of the 35 patients with DPPB underwent an emergency endoscopy, including 32 patients treated with hemostasis with clipping, one with thrombin spraying on the bleeding site, and two having no active bleeding after fasting treatment and thus were not treated endoscopically. None of them required a blood transfusion, interventional radiology or surgery, and no thromboembolic events or death occurred.

## 4 DISCUSSION

The current study showed that the overall incidence of DPPB was 1.54% after resection of colorectal polyps. The patients on antithrombotics had a significantly increased risk of bleeding than the control group who did not taking antithrombotics (2.90% vs 1.18%, *P* = 0.006). In previous studies, the incidence of DPPB varies significantly because of different definitions of DPPB and management with antithrombotics. Our results are consistent with those reported previously, with the incidence of DPPB being 1% in patients not on antithrombotics,^2^ and 1.8%‐7.0% in those who temporarily discontinue and rapidly resume antithrombotic drugs when compared with 11% in patients who have not discontinued antithrombotics.^7^ Most delayed bleeding events occur within 1‐2 weeks after endoscopic resection.^8^ And in our patients DPPB occurred at 1‐7 days after endoscopy (median 3 d).

The HB therapy subgroup had a significantly higher rate of DPPB than the control group (17.39% vs 1.18%, *P* < 0.001), and HB therapy was an independent risk factor for DPPB (OR 16.735). The guidelines have suggested that warfarin should be discontinued and replaced by HB therapy in patients at a high risk of thrombosis.^1^, ^2^, ^3^ However, the HB therapy has been reported to be an independent risk factor for DPPB and is associated with a DPPB incidence of 14.9%‐22.2% (vs 0%‐1.9% in patients who did not receive HB therapy),^9^, ^10^, ^11^ which is consistent with our results. Heparin increases the risk of DPPB in warfarin users because warfarin and heparin may be used simultaneously for several days after the procedure to achieve sufficient anticoagulation. However, in the BRIDGE trial, HB therapy increased the risk of bleeding during invasive surgery but did not significantly prevent thromboembolism.^6^ Therefore, endoscopists should strictly select patients with an indication for HB therapy in order to reduce the DPPB rate.

Use of clopidogrel was significantly associated with a higher incidence of DPPB than the control group but was not an independent risk factor for DPPB in the multivariate analysis. Two studies^12^, ^13^ indicated that clopidogrel increased the risk of DPPB, and the latter also found that clopidogrel was not an independent risk factor for DPPB. In a recent randomized controlled trial of uninterrupted clopidogrel use,^14^ the incidence of DPPB was 3.8% in the clopidogrel group and 3.6% in the placebo group. The DPPB rate in the placebo group was higher than expected, possibly due to the resumption of clopidogrel. Although clopidogrel was discontinued during the procedure in our study, the bleeding rate remains high, which might have been related to the resumption of clopidogrel at 48 hours after polypectomy.

The aspirin group had a similar DPPB rate as that of the control group. Aspirin suspension before polypectomy is not recommended in the current guidelines,^1^, ^2^, ^3^ but cessation of aspirin was documented in all patients 7 days prior to the procedure in our retrospective study. Anecdotally, Asian practitioners seem to be more prudent about antiplatelet decisions, whereas many American physicians tend to keep aspirin administration during endoscopy,^15^ although 32.5% of gastrointestinal centers instruct aspirin to be discontinued before colonoscopy in the United States.^16^ Consistent with our results, most studies indicated that aspirin did not increase the risk of DPPB,^17^, ^18^, ^19^ except in cases of large colonic lesions.^20^ DAPT has been reported to increase the risk of DPPB.^13^ In this study, the risk of DPPB was higher in the DAPT group than in the control group; however, this is not statistically significant, possibly because of the small number of patients receiving DAPT. Therefore, the decision to discontinue antiplatelet agents should be made based on the risks of thromboembolism and DPPB.

DOAC users had a trend of an increased rate of DPPB compared with the controls (3.85% vs 1.18%, *P* = 0.274). One study^21^ reported that DOAC with HB therapy increased the risk of bleeding compared with DOAC alone and did not prevent thromboembolism. In our study, none of the patients in the DOAC group received HB therapy. Compared with warfarin, DOAC have a shorter half‐life, more rapid onset of action and entail no requirement for HB therapy and perioperative INR monitoring. Therefore, DOAC are being increasingly prescribed. Due to the short history of the promotion and application of DOAC there has been no report on the correlation between DOAC and DPPB in the Chinese population. Another study^22^ has reported that DOAC confer a low risk of post‐polypectomy complications, with a DPPB rate of 0.63%. In a large multicenter study of patients with atrial fibrillation on DOAC, the safety of a temporary interruption of DOAC without HB therapy during the perioperative period was evaluated.^23^ The incidence of major bleeding events was 0.9%‐1.85% and the risk of arterial thromboembolism was 0.16%‐0.6%. Therefore, DOAC do not significantly increase the risk of DPPB. Further studies are needed to confirm the results.

We found that male sex, polyp size >1 cm and polyps located in the rectum were independent risk factors for DPPB. Polyp size has been identified as a risk factor for DPPB.^9^, ^19^, ^24^ A study^17^ reported that for each 1 mm increase in the diameter of polyps the risk of postoperative bleeding increased by 9%. Additionally, we found that male patients are more likely to experience DPPB than female patients, which is consistent with previous studies.^25^, ^26^ This may be because men have higher rates of colorectal adenomas and cancers^27^ than women and thus are more likely to undergo polyp removal during a colonoscopy. Studies have also reported that polyps in the right hemi‐colon leads to a higher DPPB rate than the lesions located in the left hemi‐colon.^9^, ^19^, ^24^ In Chinese patients, left colon and rectal neoplasms are more common^28^ than they are in Western populations. The incidence of DPPB was higher for rectal polyps, possibly because of the abundant blood vessels in the rectal mucosa, and similar results have been reported in a Japanese study.^29^ Almost half of DPPB cases do not require therapeutic intervention.^30^ These factors can be used to further stratify the risk of DPPB and identify patients likely to benefit from colonoscopy.

Although none of the patients developed thromboembolism in this study, thrombosis caused by the withdrawal of antithrombotics may more likely be fatal in high‐risk patients. Therefore, the collaborative evaluation of the risk of bleeding and thromboembolism is needed, and the appropriate treatment timing and plan must be determined.

One strength of our study is that we enrolled a relatively large number of patients on anticoagulant or antiplatelet therapy whose bleeding data were acquired by investigators do not have access to the endoscopic interventions the patients receive. There were some limitations to the study, one of which is the retrospective single‐center study design; eg, the distribution of patients was unequal among the groups, and the sample size of the DAPT and anticoagulant users was relatively small, which might have affected the liability in the statistical analyses (see the wide 95% CI in the logistic model) and undermined the value of extrapolating these results to clinical practice. However, similar results have also been noticed in studies^11^, ^29^ with sample sizes of HB therapy ranging from 20 to 45. Although these patients are relatively uncommon in clinical setting, our findings, along with those of other studies, imply that use of HB therapy warrants more caution than it has hitherto received. In addition, due to the limited number of patients undergoing cold snare polypectomy in our endoscopy center, these cases were not included in this study and studies on these patients will be conducted in the future.

## 5 CONCLUSIONS

In conclusion, patients on antithrombotics had a higher risk of DPPB than those who did not receive antithrombotics. HB and clopidogrel therapies significantly increased the incidence of DPPB. HB therapy, male sex, polyp size >1 cm, and polyps located in the rectum were independent risk factors for DPPB.

## CONFLICTS OF INTEREST

None.

## Supporting information


**Table S1.** Univariate analysis of risk factors for delayed post‐polypectomy bleeding

## References

[1] Veitch AM , Vanbiervliet G , Gershlick AH , et al. Endoscopy in patients on antiplatelet or anticoagulant therapy, including direct oral anticoagulants: British Society of Gastroenterology (BSG) and European Society of Gastrointestinal Endoscopy (ESGE) guidelines. Gut. 2016;65(3):374‐389. 26873868 10.1136/gutjnl-2015-311110 PMC4789831

[2] ASGE Standards of Practice Committee , Acosta RD , Abraham NS , et al. The management of antithrombotic agents for patients undergoing GI endoscopy. Gastrointest Endosc. 2016;83(1):3‐16. 26621548 10.1016/j.gie.2015.09.035

[3] Chan FKL , Goh KL , Reddy N , et al. Management of patients on antithrombotic agents undergoing emergency and elective endoscopy: joint Asian Pacific Association of Gastroenterology (APAGE) and Asian Pacific Society for Digestive Endoscopy (APSDE) practice guidelines. Gut. 2018;67(3):405‐417. 29331946 10.1136/gutjnl-2017-315131 PMC5868286

[4] Virani SS , Alonso A , Benjamin EJ , et al; American Heart Association Council on Epidemiology and Prevention Statistics Committee and Stroke Statistics Subcommittee . Heart disease and stroke statistics—2020 update: a report from the American Heart Association. Circulation. 2020;141(9):e139‐e596. 31992061 10.1161/CIR.0000000000000757

[5] Hee YJ , Bang CS , Baik GH , et al. Association between ischemic heart disease and colorectal neoplasm: a systematic review and meta‐analysis. Springerplus. 2016;5(1):1664. 10.1186/s40064-016-3349-0. 27730024 PMC5039141

[6] Douketis JD , Spyropoulos AC , Kaatz S , et al. Perioperative bridging anticoagulation in patients with atrial fibrillation. N Engl J Med. 2015;373(9):823‐833. 26095867 10.1056/NEJMoa1501035 PMC4931686

[7] Abraham NS . Antiplatelets, anticoagulants, and colonoscopic polypectomy. Gastrointest Endosc. 2020;91(2):257‐265. 31585125 10.1016/j.gie.2019.09.033 PMC7386094

[8] Feagins LA . Colonoscopy, polypectomy, and the risk of bleeding. Med Clin North Am. 2019;103(1):125‐135. 30466669 10.1016/j.mcna.2018.08.003

[9] Lin D , Soetikno RM , McQuaid K , et al. Risk factors for postpolypectomy bleeding in patients receiving anticoagulation or antiplatelet medications. Gastrointest Endosc. 2018;87(4):1106‐1113. 29208464 10.1016/j.gie.2017.11.024

[10] Yanagisawa N , Nagata N , Watanabe K , et al. Post‐polypectomy bleeding and thromboembolism risks associated with warfarin *vs* direct oral anticoagulants. World J Gastroenterol. 2018;24(14):1540‐1549. 29662292 10.3748/wjg.v24.i14.1540 PMC5897858

[11] Ishigami H , Arai M , Matsumura T , et al. Heparin‐bridging therapy is associated with a high risk of post‐polypectomy bleeding regardless of polyp size. Dig Endosc. 2017;29(1):65‐72. 27368065 10.1111/den.12692

[12] Feagins LA , Iqbal R , Harford WV , et al. Low rate of postpolypectomy bleeding among patients who continue thienopyridine therapy during colonoscopy. Clin Gastroenterol Hepatol. 2013;11(10):1325‐1332. 23403011 10.1016/j.cgh.2013.02.003

[13] Singh M , Mehta N , Murthy UK , Kaul V , Arif A , Newman N . Postpolypectomy bleeding in patients undergoing colonoscopy on uninterrupted clopidogrel therapy. Gastrointest Endosc. 2010;71(6):998‐1005. 20226452 10.1016/j.gie.2009.11.022

[14] Chan FKL , Kyaw MH , Hsiang JC , et al. Risk of postpolypectomy bleeding with uninterrupted clopidogrel therapy in an industry‐independent, double‐blind, randomized trial. Gastroenterology. 2019;156(4):918‐925.e1. 30518511 10.1053/j.gastro.2018.10.036

[15] Lee SY , Tang SJ , Rockey DC , et al. Managing anticoagulation and antiplatelet medications in GI endoscopy: a survey comparing the East and the West. Gastrointest Endosc. 2008;67(7):1076‐1081. 18384789 10.1016/j.gie.2007.11.037

[16] Robbins R , Tian C , Singal A , Agrawal D . Periprocedural management of aspirin during colonoscopy: a survey of practice patterns in the United States. Gastrointest Endosc. 2015;82(5):895‐900. 25975531 10.1016/j.gie.2015.03.1976

[17] Sawhney MS , Salfiti N , Nelson DB , Lederle FA , Bond JH . Risk factors for severe delayed postpolypectomy bleeding. Endoscopy. 2008;40(2):115‐119. 18253906 10.1055/s-2007-966959

[18] Yousfi M , Gostout CJ , Baron TH , et al. Postpolypectomy lower gastrointestinal bleeding: potential role of aspirin. Am J Gastroenterol. 2004;99(9):1785‐1789. 15330919 10.1111/j.1572-0241.2004.30368.x

[19] Choung BS , Kim SH , Ahn DS , et al. Incidence and risk factors of delayed postpolypectomy bleeding: a retrospective cohort study. J Clin Gastroenterol. 2014;48(9):784‐789. 24231934 10.1097/MCG.0000000000000027

[20] Metz AJ , Bourke MJ , Moss A , Williams S , Swan M , Byth K . Factors that predict bleeding following endoscopic mucosal resection of large colonic lesions. Endoscopy. 2011;43(6):506‐511. 21618150 10.1055/s-0030-1256346

[21] Nagata N , Yasunaga H , Matsui H , et al. Therapeutic endoscopy‐related GI bleeding and thromboembolic events in patients using warfarin or direct oral anticoagulants: results from a large nationwide database analysis. Gut. 2018;67(10):1805‐1812. 28874418 10.1136/gutjnl-2017-313999 PMC6145295

[22] Yu JX , Oliver M , Lin J , et al. Patients prescribed direct‐acting oral anticoagulants have low risk of postpolypectomy complications. Clin Gastroenterol Hepatol. 2019;17(10):2000‐2007.e3. 30503964 10.1016/j.cgh.2018.11.051 PMC6541555

[23] Douketis JD , Spyropoulos AC , Duncan J , et al. Perioperative management of patients with atrial fibrillation receiving a direct oral anticoagulant. JAMA Intern Med. 2019;179(11):1469‐1478. 31380891 10.1001/jamainternmed.2019.2431 PMC6686768

[24] Kim JH , Lee HJ , Ahn JW , et al. Risk factors for delayed post‐polypectomy hemorrhage: a case‐control study. J Gastroenterol Hepatol. 2013;28(4):645‐649. 23369027 10.1111/jgh.12132

[25] Niikura R , Yasunaga H , Yamada A , et al. Factors predicting adverse events associated with therapeutic colonoscopy for colorectal neoplasia: a retrospective nationwide study in Japan. Gastrointest Endosc. 2016;84(6):971‐982.e6. 27189658 10.1016/j.gie.2016.05.013

[26] Rabeneck L , Paszat LF , Hilsden RJ , et al. Bleeding and perforation after outpatient colonoscopy and their risk factors in usual clinical practice. Gastroenterology. 2008;135(6):1899‐1906.e1. 18938166 10.1053/j.gastro.2008.08.058

[27] Hoffmeister M , Schmitz S , Karmrodt E , et al. Male sex and smoking have a larger impact on the prevalence of colorectal neoplasia than family history of colorectal cancer. Clin Gastroenterol Hepatol. 2010;8(10):870‐876. 20670694 10.1016/j.cgh.2010.07.004

[28] Chen H , Li N , Ren J , et al; Group of Cancer Screening Program in Urban China (CanSPUC) . Participation and yield of a population‐based colorectal cancer screening programme in China. Gut. 2019;68(8):1450‐1457. 30377193 10.1136/gutjnl-2018-317124

[29] Kishida Y , Hotta K , Imai K , et al. Risk analysis of colorectal post‐polypectomy bleeding due to antithrombotic agent. Digestion. 2019;99(2):148‐156. 30179871 10.1159/000490791

[30] Rodríguez de Santiago E , Hernández‐Tejero M , Rivero‐Sánchez L , et al. Management and outcomes of bleeding within 30 days of colonic polypectomy in a large, real‐life multicenter cohort study. Clin Gastroenterol Hepatol. 2021;19(4):732‐742.e6. 32272252 10.1016/j.cgh.2020.03.068

