# Supplementary material for: Incidence and risk factors of colorectal delayed post‐polypectomy bleeding in patients taking antithrombotics
Source: J Dig Dis. 2021 Aug 16;22(8):481–7. doi: 10.1111/1751-2980.13034 (PMC9291470; doi:10.1111/1751-2980.13034)
Supplement: Supplementary file 1 — Table S1. Univariate analysis of risk factors for delayed post‐polypectomy bleeding [file CDD-22-481-s001.docx]

**Supplement Table 1.** Univariate analysis of risk factors for delayed postpolypectomy bleeding

| **Variables** | **Odds ratio** | **Unadjusted**  **95% CI** | ***P* value^a^** |
| --- | --- | --- | --- |
| Age (years) | 0.978 | 0.952-1.005 | 0.113 |
| Gender (male vs female) | 4.571 | 1.608-12.994 | 0.004 |
| Comorbidity |  |  |  |
| Hypertension (yes vs no) | 0.859 | 0.435-1.699 | 0.663 |
| Diabetes mellitus (yes vs no) | 0.427 | 0.130-1.401 | 0.160 |
| Coronary heart disease (yes vs no) | 1.674 | 0.725-3.868 | 0.228 |
| PCI (yes vs no) | 2.194 | 0.762-6.312 | 0.145 |
| CABG (yes vs no) | 2.955 | 0.387-22.551 | 0.296 |
| Peripheral arterial disease (yes vs no) | 5.020 | 0.639-39.467 | 0.125 |
| Malignancy (yes vs no) | 0.457 | 0.062-3.363 | 0.442 |
| Antithrombotics |  |  |  |
| Control | 1.000 (reference) |  |  |
| Aspirin | 1.091 | 0.372-3.200 | 0.874 |
| Clopidogrel | 4.308 | 1.444-12.851 | 0.009 |
| DAPT | 3.231 | 0.419-24.924 | 0.261 |
| Warfarin | NA | NA | 0.999 |
| Warfarin + heparin bridge therapy | 17.684 | 5.539-56.461 | *<*0.001 |
| DOACs  Polyp factors | 3.360 | 0.435-25.959 | 0.245 |
| Polyp number ≥ 3 (yes vs no) | 1.050 | 0.520-2.121 | 0.892 |
| Polyp size >1 cm (yes vs no) | 7.235 | 3.270-16.005 | <0.001 |
| Pedunculated shape (yes vs no) | 5.858 | 2.956-11.610 | <0.001 |
| LST (yes vs no) | 0.796 | 0.189-3.346 | 0.755 |
| Polyp located in right side colon (yes vs no) | 0.615 | 0.287-1.318 | 0.211 |
| Polyp located in the rectum (yes vs no) | 8.946 | 4.349-18.402 | <0.001 |

**^a^**Univariate logistic regression analysis.

Abbreviations: CABG, [coronary artery bypass grafting](https://www.ncbi.nlm.nih.gov/pubmed/30094215); CI, confidence interval; DAPT, dual antiplatelet therapy; DOAC, direct oral anticoagulants; LST, laterally spreading tumor; NA, not applicable; PCI, percutaneous coronary intervention.
